# Real-world treatments and outcomes of chronic graft-versus-host disease after allogeneic hematopoietic stem cell transplantation: a Japanese claims analysis

**DOI:** 10.1007/s12185-026-04228-9

**Published:** 2026-05-27

**Authors:** Takero Shindo, Mami Shindo

**Affiliations:** 1 Department of Hematology and Oncology, Hiroshima University Research Institute for Radiation Biology and Medicine, 1-2-3 Kasumi, Minami-ku, Hiroshima, 734-8553 Japan; 2 Meiji Seika Pharma Co., Ltd., Tokyo, Japan

**Keywords:** Chronic GVHD, Allogeneic hematopoietic cell transplantation, Steroid, Adverse event, Real-world data

## Abstract

Immunosuppressants can be used to treat chronic graft-versus-host disease (cGVHD) following allogeneic hematopoietic cell transplantation. However, real-world retrospective studies on practical regimens and health outcomes remain limited. To identify challenges associated with systemic treatment of cGVHD, we analyzed anonymized Japanese medical insurance claims data. Among 1090 patients with cGVHD, 81% received systemic treatment with immunosuppressive drugs. The most common initial regimen was a combination of tacrolimus and steroids. Second-line treatment was provided in 44% of patients including the addition of steroids, calcineurin inhibitors, or mycophenolate mofetil, or switching to another drug class. Third-line treatment was provided in 23% and steroid monotherapy was the most commonly used. Immunosuppressive treatment was temporarily discontinued in 79% of patients but was re-initiated in approximately a half of them. Young recipients (< 15 years) and umbilical cord blood recipients had lower rates of treatment re-initiation and conferred lower mortality. Various complications were observed after transplantation but most of them gradually resolved, with the exception of chronic bone, eye, and respiratory system disorders. The immunocompromised state of these patients was also reflected in the frequent use of anti-infective agents. The adverse effects associated with long-term steroid use pose a clinical challenge, highlighting the need for alternative treatments.

**Supplementary Information:**

The online version contains supplementary material available at https://doi.org/10.1007/s12185-026-04228-9.

## Introduction

Chronic graft-versus-host disease (cGVHD) is a major complication of allogeneic hematopoietic cell transplantation (HCT). Multi-organ inflammatory and fibrotic changes cause increased morbidity, higher late mortality, and residual disorders that significantly impair quality-of-life [1]. For systemic treatment of cGVHD, low-dose prednisolone is typically recommended as first-line therapy, sometimes in combination with calcineurin inhibitors [2, 3]. However, prolonged use of immunosuppressive agents can increase the risk of infections and secondary malignancies [4, 5]. Despite the clinical importance, few real-world studies have focused on the practical selection of treatments, associated adverse events, and complications in cGVHD patients. In this study, we analyzed medical records of patients with cGVHD using Japanese insurance claims data to clarify challenges related to systemic treatment. The analysis was further stratified by recipient age and donor cell source.

## Materials and methods

### Study design and data source

This study retrospectively analyzed an anonymized Japanese medical insurance claims database maintained by JMDC Inc. (Tokyo, Japan). The dataset included medical records of 14 million patients, incorporating ICD-10 code-based clinical diagnoses, standardized medical procedures, and Anatomical Therapeutic Chemical (ATC) classification-based prescriptions [6]. As the study used de-identified data, the requirement for informed consent was waived. The study was conducted in accordance with the principles of the Declaration of Helsinki and approved by the Ethics Committee of the Kyoyukai Riverside Clinic (Sapporo, Japan; approval number: RSC-2406RB01).

### Definition of cGVHD

**Fig. 1:**
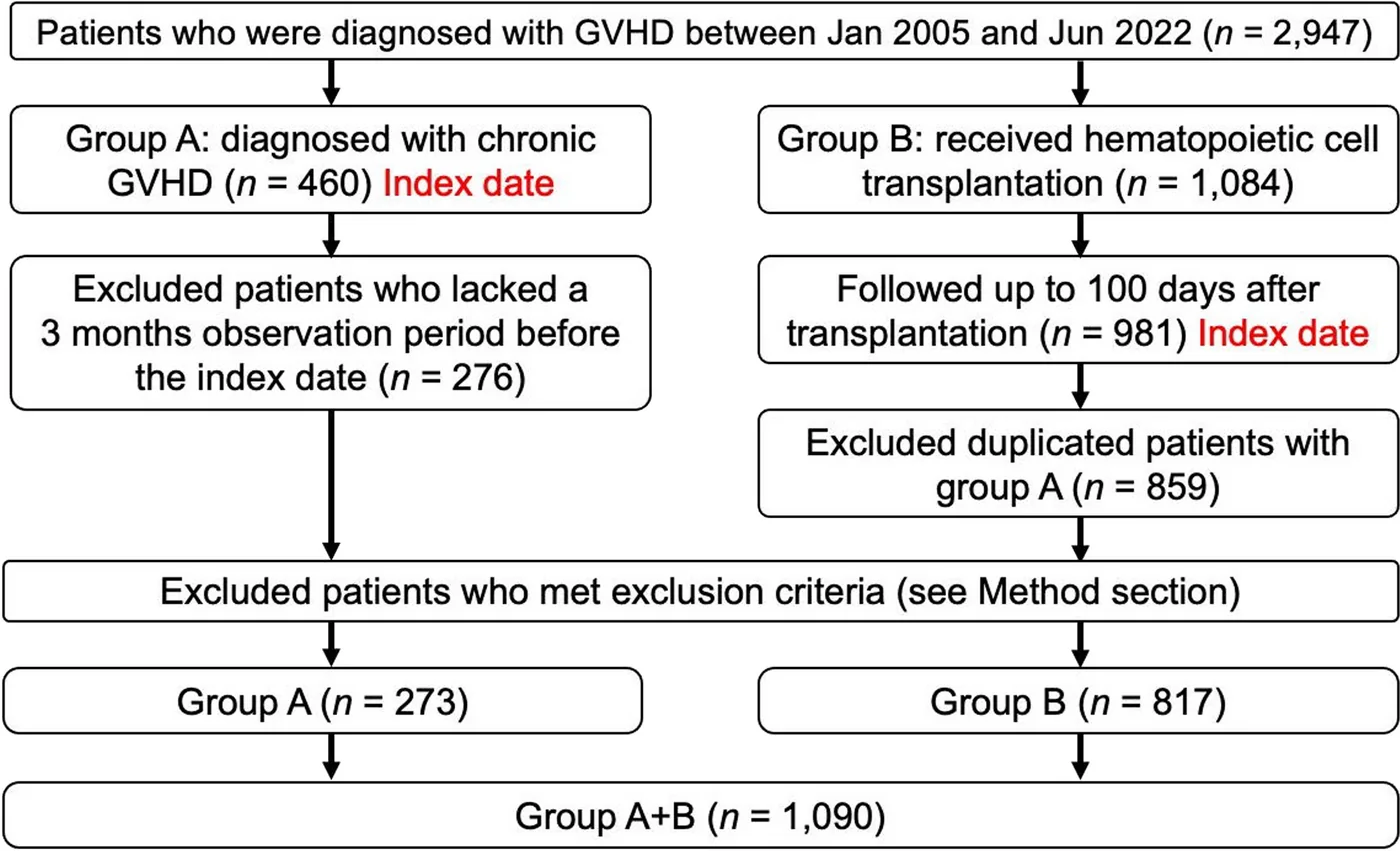
Flowchart of patient selection

An outline of the patient selection process is shown in Fig. 1. First, graft-versus-host disease (GVHD) was defined by ICD-10 code T860 ‘complications of bone marrow transplant’ and/or receipt code (Supplementary Table 1), and individuals diagnosed with GVHD between January 2005 and June 2022 were extracted (*n* = 2,947). Patients with a confirmed diagnosis of cGVHD were assigned to Group A (*n* = 460), and the date of diagnosis was set as the index date. Patients in group A who lacked a look-back period of at least 3 months prior to the index date were excluded. Patients who underwent HCT were assigned to Group B (*n* = 1,084). Those who developed cGVHD after a 100-day follow-up period were included (*n* = 981), with the date marking day 100 defined as the index date. Patients duplicated between Groups A and B were excluded from Group B. All the patients had confirmed diagnoses of hematologic disorders in the claims. The following exclusion criteria were applied to both groups:

1. Patients who received only autologous transplantation prior to the index date (*n* = 34 in Group B)
2. Patients who received autologous transplantation after allogeneic transplantation (*n* = 3, group B)
3. Patients not diagnosed with GVHD after their most recent allotransplantation (*n* = 1 and 4 in Groups A and B, respectively).
4. Patients diagnosed with GVHD unrelated to HCT (*n* = 4 and 1 in groups A and B, respectively).

A total of 1,090 patients were included in the cGVHD population (273 in Group A and 817 in Group B).

### Observation and measures

Patient characteristics (age and gender) were recorded at the index date. For patients who underwent HCT, data on the year of transplantation, stem cell source (bone marrow, peripheral blood, or umbilical cord blood), and donor type (related or unrelated) were recorded. Medical events, including ICD-10-based clinical diagnoses, standardized medical procedures, and ATC-classified prescriptions, were extracted. Detailed definitions are provided in Supplementary Tables S2–S4.

For cGVHD treatment, the initially prescribed systemic (oral or injectable) immunosuppressive regimen (single or multiple agents) was defined as the first-line treatment. Treatment duration was calculated from prescription records and summarized in the categorized population.

If the regimen was changed to another drug or a combination therapy, it was considered a new treatment regimen. However, if the new agents were initiated within 28 days of starting the initiation of the ongoing regimen, they were regarded as part of the same treatment. A treatment that was suspended for 14 days or more later re-initiated was also considered a new treatment regimen, even if the same agents were resumed.

Steroid doses were normalized to prednisolone equivalents (Supplementary Table S3) based on established conversion factors [7] and recorded accordingly. Separate analyses were performed for cases in which treatment was temporally suspended (≥ 14 days) and re-initiated. Complications (ICD-10 codes), prescriptions for anti-infective agents (ATC code J) and death were monitored after the index date (presumed onset of cGVHD). These outcomes were recorded as long as the patient remained in the dataset.

## Data analysis

Categorical variables are summarized as the number of patients in the population (*N*), the number of patients who experienced the events (*n*), and the corresponding percentage. Continuous data (age, dosage duration, and doses) are presented as the median (with or without interquartile range [IQR]) and/or the mean with standard deviation (SD). In several analyses, the population was stratified by age (< 15, 15–54, ≥ and 55 years) or by HCT donor source. All data processing was performed using PostgreSQL 8.0.2 on an i686-pc-linux-gnu system.

## Results

### Patient characteristics

Patient and transplantation characteristics are summarized in Table 1. In this retrospective cohort of 1,090 patients with cGVHD, the median age was 45 years, and 701 (64.3%) were male. The most common primary disease was myeloid leukemia (441 cases, 40.5%), of which 373 cases were acute myeloid leukemia, followed by lymphocytic leukemia (216 cases, 19.8%), myelodysplastic syndrome (212 cases, 19.4%), and non-Hodgkin lymphoma (183 cases, 16.8%). Transplant sources included bone marrow (322 cases, 29.5%), peripheral blood (255 cases, 23.4%), and umbilical cord blood (355 cases, 32.6%). The median observation period from the index date (i.e., first diagnosis of cGVHD) was 16 months, with a mean ± SD of 27.5 ± 30.5 months, reflecting wide variability in observation period.

**Table 1:** Baseline characteristics of patients

Characteristic		cGVHD population (*N* = 1090) %	
Age	median	45	
	mean ± SD	40.1 ± 18.4	
	< 15	138	12.7
	15–54	665	61.0
	≥ 55	287	26.3
Sex	male	701	64.3
	female	389	35.7
Primary disease^*^	Hodgkin lymphoma	6	0.6
	non-Hodgkin lymphoma	183	16.8
	myeloma and primary macroglobulinemia	19	1.7
	lymphocytic leukemia	216	19.8
	adult T-cell leukemia	29	2.7
	myeloid leukemia	441	40.5
	myelodysplastic syndrome	212	19.4
	myeloproliferative disorder	18	1.7
	aplastic anemia	54	5.0
	others	118	10.8
	unknown	38	3.5
HCT year^**^	before 2017	344	31.6
	2017 or after	588	53.9
HCT source^**^	bone marrow	322	29.5
	peripheral blood	255	23.4
	umbilical cord blood	355	32.6
Donor^**^	related	324	29.7
	unrelated	129	11.8
	unknown	479	43.9
Observation period	median	16 months	
	mean ± SD	27.5 ± 30.5 months	
	< 3 months	129	11.8
	3–6 months	143	13.1
	6–12 months	179	16.4
	12–24 months	219	20.1
	24–60 months	261	24.0
	≥ 60 months	159	14.6

^*^There were several cases with multiple diagnoses

^**^In 158 (14.5%) cases, there was no record of the timing, source, or donor of HCT

When patient characteristics were stratified by recipient age (Supplementary Table S5), notable differences in primary disease distribution were observed: Myeloid leukemia was less frequent in the young group, while myelodysplastic syndrome was more prevalent in the elderly group. Cord blood transplantation was preferably selected in the young group (42.0%), and the observation period was markedly longer than that in the adult and elderly groups.

### Treatment regimens

As shown in Table 2, a combination of tacrolimus (TAC) and steroids was the most common primary treatment regimen, used in 273 patients (30.8%) with a median treatment duration of 105 days. Steroids alone and TAC alone were used as the first-line therapy in 179 (20.2%) and 244 (27.6%) patients, with median durations of 15 and 50 days, respectively. Cyclosporin (CSP) was selected far less frequently than TAC, either alone or in combination with steroids, although the treatment duration was similar to that of TAC. Use of mycophenolate mofetil (MMF) was limited and mostly observed in combination with steroids (and TAC). In age-stratified analysis of the first-line therapy (Supplementary Table S6), the TAC-steroid combination was more frequently used in the young group (42.1%), whereas steroids alone (12.1%) or CSP-steroid combination (1.9%) were less commonly selected in this group.

**Table 2:** Immunosuppressants used as the first, second and third treatments

Prescription	First treatment			Second treatment			Third treatment		
	*N* = 885 (81%)^*^			*N* = 482 (44%)^*^			*N* = 251 (23%)^*^		
	*n*	%	Days^**^	*n*	%	Days^**^	*n*	%	Days^**^
TAC + Steroid	273	30.8	105	173	35.9	125	56	22.3	109
TAC	244	27.6	50	47	9.8	29	29	11.6	44
Steroid	179	20.2	15	103	21.4	30	94	37.5	29
CSP	74	8.4	66.5	18	3.7	16	11	4.4	18
CSP + Steroid	58	6.6	120	48	10.0	154.5	17	6.8	84
TAC + Steroid + MMF	27	3.1	224	30	6.2	230	15	6.0	185
Steroid + MMF	5	0.6	17	7	1.5	31	7	2.8	282
Others	25	2.8	–	56	11.6	–	22	8.7	–

^*^The number of patients (and the percentage) who received the treatment regimen at each stage

^**^Median days of continuous dosing are shown

*TAC* tacrolimus, *CSP* cyclosporin *MMF* mycophenolate mofetil

**Fig. 2:**
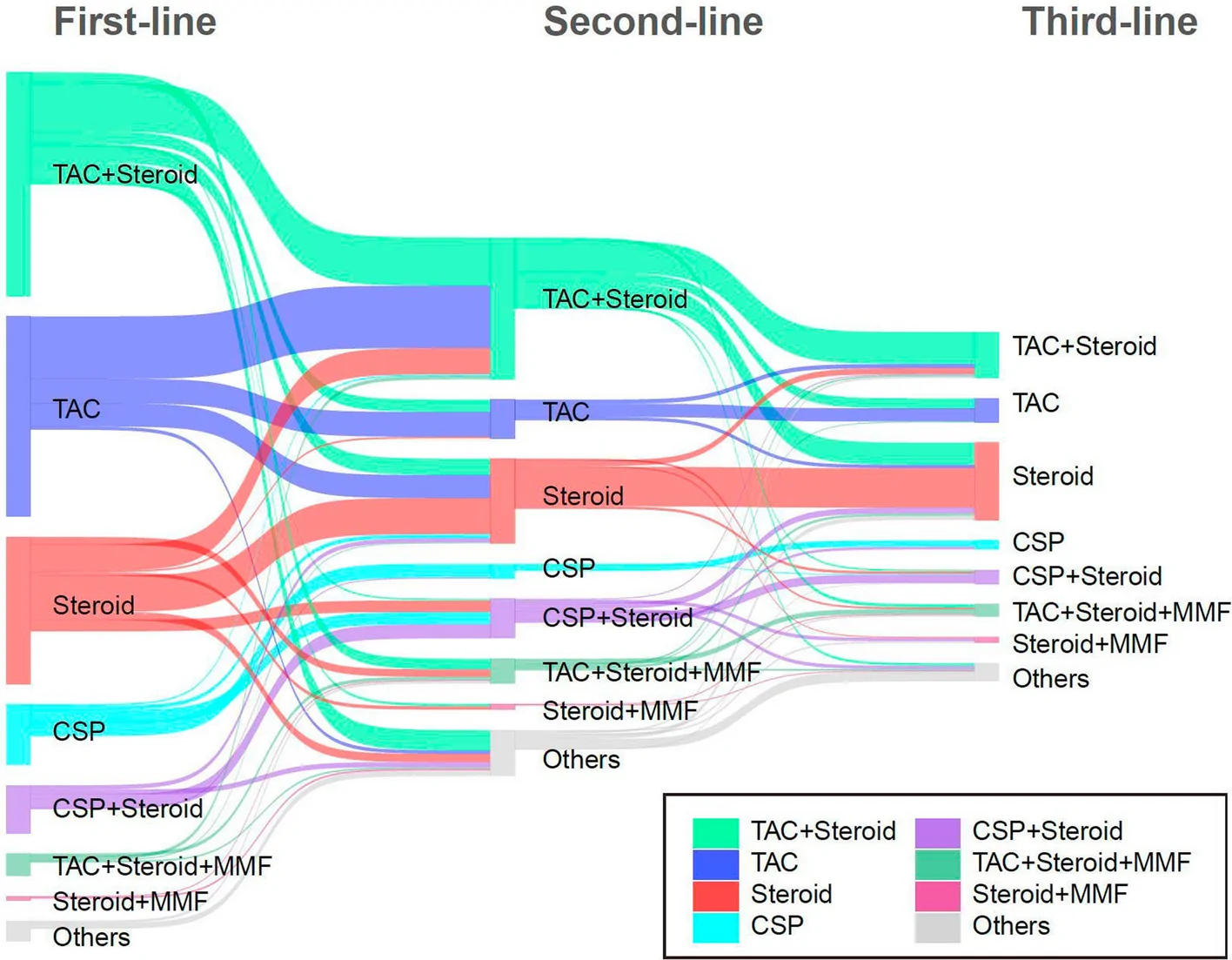
Sankey diagram for the lines of treatment in cGVHD

Second-line treatment was administered in approximately a half of the patients who received first-line treatment—482 of 885 (44.2%) who received any primary regimen, including 137 of 273 (50.2%) who received the most common primary regimen (TAC and steroids). The TAC-steroid combination remained the most common second-line regimen, used in 173 cases (35.9%). As evident in the Sankey diagram (Fig. 2), addition of steroids was preferred in first-line treatment with TAC or CSP alone. For third-line treatment, steroids were the most common, used in 94 (37.5%) cases. Overall, steroid use increased progressively across treatment lines. The combination of TAC, steroids, and MMF was associated with prolonged treatment duration irrespective of treatment stage. Recently approved kinase inhibitors for cGVHD, ibrutinib and ruxolitinib, were prescribed in only 23 and 12 patients, respectively, across all three lines of treatment. These therapies are included under the ‘Others’ category in Table 2.

Regarding discontinuation and intermission of immunosuppressive treatments (Table 3), among the 885 (81.2% of the total cGVHD cohort) who received any immunosuppressant, treatment was discontinued for 14 days or more at least once in 703 (79.4%) cases. However, treatment was reintroduced after a median of 29 days in 355 (50.4%) patients. Stratified analysis indicated that patients under 15 years of age were less likely to receive immunosuppressive therapy overall and more likely to successfully discontinue treatment compared to older patients. Among cord blood recipients, the rate of treatment resumption was also lower.

**Table 3:** Continuation of immunosuppressants in cGVHD patients

Age	All ages		Age < 15		Age 15–54		Age ≥ 55	
	*N* = 1,090		*N* = 138		*N* = 665		*N* = 287	
	*n*	%	*n*	%	*n*	%	*n*	%
Prescribed any immunosuppressant^*^	885	81.2	107	77.5	547	82.3	231	80.5
	(100)		(100)		(100)		(100)	
Dosing suspended	703	(79.4)	93	(86.9)	437	(79.9)	173	(74.9)
Dosing resumed	355	(50.4)	38	(40.9)	234	(53.5)	83	(48.0)
Intermission (median days)	29		28		29		27	
HCT Source^**^			Bone marrow		Peripheral blood		Cord blood	
			*N* = 322		*N* = 255		*N* = 355	
			*n*	%	*n*	%	*n*	%
Prescribed any immunosuppressant^*^			289	89.8	220	86.3	288	81.1
			(100)		(100)		(100)	
Dosing suspended			235	(81.3)	177	(80.5)	220	(76.4)
Dosing resumed			125	(53.2)	97	(54.8)	86	(39.1)
Intermission (median days)			29		26		29.5	

^*^The number and percentage of patients who received any immunosuppressing treatment

^**^In 158 (14.5%) cases, there were no record for HCT

**Fig. 3:**
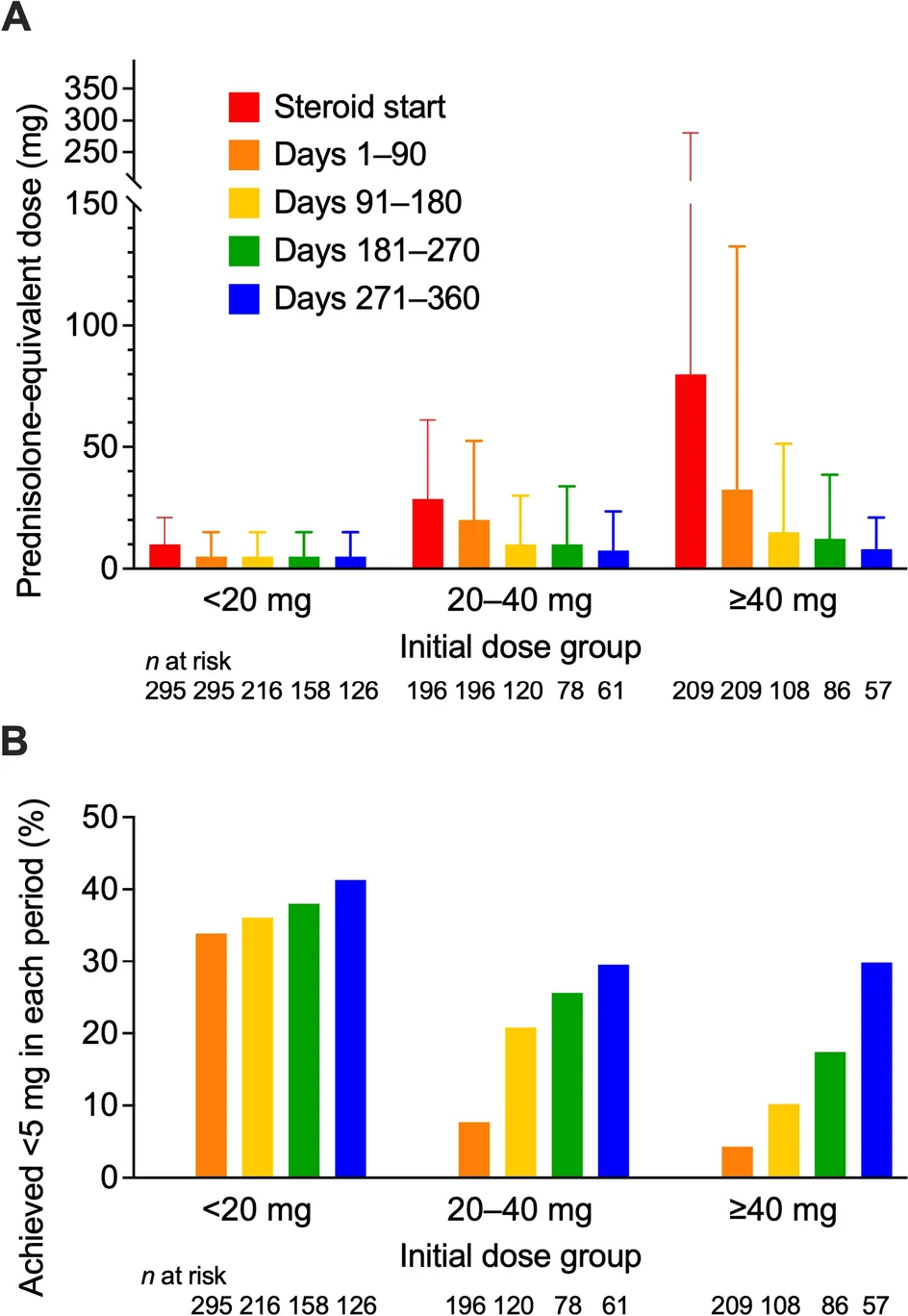
Time course of steroid dose given to cGVHD patients. **A**, Patients were grouped according to the initial prednisolone-equivalent dose, and the last daily dose during the indicated 90-day period was summarized as the median with upper quartile range. **B**, The proportion of patients who achieved in reducing the steroid dose under 5 mg is shown in each period. The numbers of cases (*n* at risk) are shown at the bottom

Analysis of steroid dosing (Fig. 3) revealed that patients initially treated with lower doses of steroids were more likely to achieve a dose of < 5 mg/day within one year. Among 295 patients who began treatment with < 20 mg/day steroid, 126 (42.7%) reduced their dose to < 5 mg/day after 360 days. Meanwhile, in patients initially receiving more steroids, the success rate in reducing the dose was low: 61of 196 patients (31.1%) who began with 20–40 mg, and 57 out of 209 patients (27.2%) who started with ≥ 40 mg, achieved this level of dose reduction.

### Complications

As shown in Table 4, a broad range of complications was recorded as diagnosis codes among cGVHD patients. The frequently-raised diseases included cytomegalovirus (CMV) infection (64.1%), diabetes mellitus (58.9%), and hypogammaglobulinemia (52.5%), all of which affected a majority of the population. Even focusing on newly onset complications raised after the index date (cGVHD phase), substantial patients suffered from those complications, though the incidence rates declined. On the other hand, other complications such as osteoporosis, dry eye and respiratory disorders (chronic obstructive pulmonary disease and bronchial asthma) became dominant as delayed-onset complications. Growth hormone deficiency was also a characteristic, chronic complication among younger patients. Differences in the incidence rates among age groups became obscure in the case of diabetes mellitus.

**Table 4:** Complications observed in cGVHD patients

Complication	Period	All ages				Age < 15				Age 15–54				Age ≥ 55			
		*N* = 1,090				*N* = 138				*N* = 665				*N* = 287			
		All^*^		Post^**^		All		Post		All		Post		All		Post	
		*n*	%	*n*	%	*n*	%	*n*	%	*n*	%	*n*	%	*n*	%	*n*	%
Cytomegalovirus infection		699	(64.1)	103	(9.4)	67	(48.6)	14	(4.3)	426	(64.1)	51	(7.7)	206	(71.8)	38	(13.2)
Diabetes mellitus		642	(58.9)	113	(10.4)	25	(18.1)	15	(10.9)	411	(61.8)	80	(12.0)	206	(71.8)	38	(13.2)
Hypogammaglobulinemia		572	(52.5)	100	(9.2)	117	(84.8)	3	(2.2)	323	(48.6)	63	(9.5)	132	(46.0)	34	(11.8)
Dry eye		480	(44.0)	171	(15.7)	34	(24.6)	13	(9.4)	322	(48.4)	122	(18.3)	124	(43.2)	36	(12.5)
Osteoporosis		442	(40.6)	197	(18.1)	32	(23.2)	21	(15.2)	295	(44.4)	136	(20.5)	115	(40.1)	40	(13.9)
Thrombocytopenia		436	(40.0)	36	(3.3)	74	(53.6)	3	(2.2)	259	(38.9)	19	(2.9)	103	(35.9)	14	(4.9)
Chronic obstructive pulmonary disease		395	(36.2)	138	(12.7)	41	(29.7)	20	(14.5)	248	(37.3)	90	(13.5)	106	(36.9)	28	(9.8)
Erythrocytopenia (anemia)		377	(34.6)	56	(5.1)	64	(46.4)	8	(2.9)	220	(33.1)	30	(4.5)	93	(32.4)	18	(6.3)
Bronchial asthma		377	(34.6)	129	(11.8)	79	(57.2)	23	(16.7)	229	(34.4)	83	(12.5)	69	(24.0)	23	(8.0)
Neutropenia		337	(30.9)	38	(3.5)	35	(25.4)	6	(4.3)	209	(31.4)	21	(3.2)	93	(32.4)	11	(3.8)
Pancytopenia		142	(13.0)	22	(2.0)	29	(21.0)	4	(2.9)	82	(12.3)	15	(2.3)	31	(10.8)	3	(1.0)
Leukocytopenia		43	(3.9)	9	(0.8)	5	(3.6)	2	(1.4)	28	(4.2)	4	(0.6)	10	(3.5)	3	(1.0)
Growth hormone deficiency		19	(1.7)	15	(1.4)	14	(10.1)	11	(8.0)	5	(0.8)	4	(0.6)	0	(0.0)	0	(0.0)

^*^Number and proportion of patients who received the diagnosis of complication throughout the observation period

^**^Number and proportion of patients excluding who received the diagnosis initially before the month including the index date

The immunocompromised status of cGVHD patients was further reflected by the widespread use of anti-infective agents during treatment (Table 5). 94.0% of patients received at least one such agent in total. The most commonly used reagents were anti-herpes virus agents (83.4%), systemic antifungal agents (66.5%), and trimethoprim preparations (62.7%), typically administered as preventive or preemptive therapy. Except for anti-herpesvirus agents, these agents, including immunoglobulin preparations, were used more frequently for young patients.

**Table 5:** Anti-infective agents prescribed for cGVHD patients

Prescription	All ages		Age < 15		Age 15–54		Age ≥ 55	
	*N* = 1,090		*N* = 138		*N* = 665		*N* = 287	
	*n*	%	*n*	%	*n*	%	*n*	%
Any anti-infective agent	1,025	94.0	132	95.7	628	94.4	265	92.3
Anti-herpesvirus agents	909	83.4	114	82.6	553	83.2	242	84.3
Systemic antifungal agents	725	66.5	88	63.8	436	65.6	201	70.0
Trimethoprim preparations	683	62.7	103	74.6	407	61.2	173	60.3
Oral fluoroquinolones	572	52.5	27	19.6	383	57.6	162	56.4
Intravenous cephalosporins	452	41.5	52	37.7	269	40.5	131	45.6
Intravenous immunoglobulins	377	34.6	69	50.0	209	31.4	99	34.5
Penem antibiotics	359	32.9	40	29.0	210	31.6	109	38.0
Macrolide antibiotics	356	32.7	68	49.3	222	33.4	66	23.0
Oral cephalosporins	327	30.0	82	59.4	198	29.8	47	16.4
Intravenous broad-spectrum penicillins	271	24.9	27	19.6	152	22.9	92	32.1
Glycopeptide antibiotics	233	21.4	26	18.8	131	19.7	76	26.5

### Mortality

**Fig. 4:**
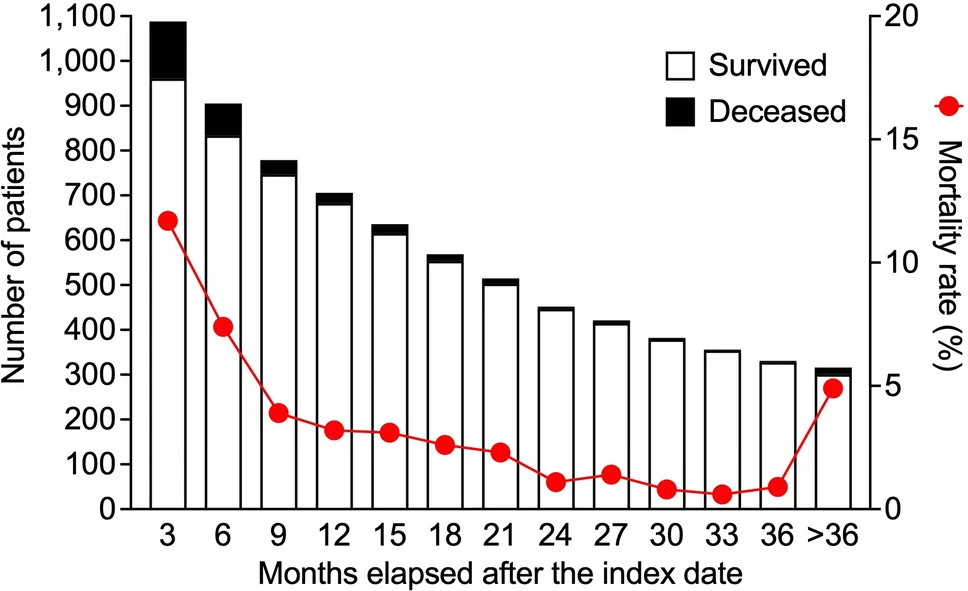
Number of cGVHD patients and the mortality rate

As shown in Table 6, 334 patients (30.6%) died during the observation period. Mortality rates were highest in the elderly group and lowest in the young group, although the median lifespans were similar. Patients who received cord blood transplants tended to have longer survival than those who received other graft types. Time course analysis (Fig. 4, Supplementary Table S7) revealed that 127 (12%) and 253 (23%) patients died within the first 3 months and 1 year, respectively, following the index date. Notably, in the first 3 months, the mortality rate exceeded 10%. The use of immunosuppressants may have contributed to early mortality in cGVHD patients (Supplementary Table S7).

**Table 6:** Deceased cGVHD patients

Age	All		Age < 15		Age 15–54		Age ≥ 55	
	*n*	%	*n*	%	*n*	%		
Initial cohort	1090		138		665		287	
Deceased	334	30.6	21	15.2	201	30.2	112	39.0
Lifespan^*^	4 (1–11)		4 (2–9)		4 (1–13)		4 (1–9)	
HCT source			Bone marrow		Peripheral blood		Cord blood	
			*n*	%	*n*	%	*n*	%
Initial cohort			322		255		355	
Deceased			112	34.8	106	41.6	90	25.4
Lifespan^*^			3 (1–12)		4 (1.3–9)		5 (2–10)	

^*^Median with IQR (months)

## Discussion

This study demonstrates that systemic immunosuppressive therapy for cGVHD predominantly involved steroids, with or without calcineurin inhibitors, and was frequently accompanied by modifications in the selection of agents, as previously reported in US claims data [8]. A recent nationwide registry-based research in Japan have captured detailed clinical and transplant-related GVHD data [9]. The present study collects real-world prescription data from insurance claims and clarified a high rate of temporal discontinuation/re-initiation and dose tapering. Importantly, our data showed that cGVHD was strongly associated with various complications, which can be attributed to both the disease itself and long-term use of immunosuppressive agents. Mortality exceeded 10% during the first 3 months following the index date and remained high despite early treatment initiation, suggesting that conventional immunosuppressive therapy may provide suboptimal control of cGVHD manifestations. We were unable to evaluate the effectiveness of recently approved kinase inhibitors due to the limited use of ibrutinib and ruxolitinib in our cohort, which consisted of cases recorded prior to June 2022.

Further stratification analysis by age and HCT source suggested that young (< 15 years) patients and those who received umbilical cord blood had higher rates of successful immunosuppressant withdrawal and higher survival rates. These observations are consistent with prior studies reporting lower incidence and severity of cGVHD in cord blood recipients [10–13]. Age group differences may reflect underlying factors, including the primary disease responsible for HCT, the source of donor cells, and severity of cGVHD. The lower use of steroids in young patients may be attributed to concerns about long-term steroid use.

Our findings also highlight the high burden of complications in cGVHD patients. The immunocompromised state of cGVHD patients may be related to the frequent use of preemptive anti-infective agents. Although causal relationships between specific agents and complications cannot be confirmed in this study, long-term steroid use is likely a major contributor to increased susceptibility to infections, diabetes mellitus and osteoporosis [7]. Calcineurin inhibitors induce diabetes mellitus [14]. Notably, younger patients experienced higher rates of hypogammaglobulinemia, thrombocytopenia, anemia, bronchial asthma, and growth hormone deficiency. Since steroids are typically used to treat bronchial asthma, these results highlight the limitations of conventional steroid-based therapy and emphasize the importance of hematological and endocrinological monitoring in young HCT recipients.

Infections remain one of the most serious complications in the treatment of cGVHD, and are a leading cause of death within a short period after HCT [2]. Given that the cases of CMV infection diagnosed after the index dates were not so many, it is unclear that cGVHD was associated with it. However, it is difficult to clearly visualize the association between cGVHD and CMV infection depending on claims data. Therefore, we simply noted the frequency of the cases with CMV infection in this study, which should be further explored. But it is still important that some cGVHD patients developed CMV infection and needed medication even in chronic phase. Our findings point to a persistent unmet need for systemic treatments for cGVHD. The adverse events associated with long-term steroid use pose a major clinical challenge. Development of safer treatments that reduce these adverse events is urgently needed. Therapeutic agents with a low risk of infection are expected to be developed in the future, and it is important to consider switching from immunosuppressive agents as early as possible.

This study utilized insurance claims data, and caution is warranted when interpreting and generalizing the results. The National Institutes of Health criteria [15] were not used to specify cGVHD due to difficulties of extracting detailed symptoms from the data. The dataset was limited to contracted medical institutions and may not fully reflect broader clinical practice (i.e., not random sampling). Diagnoses were based on routinely collected health data and included diagnoses made for insurance claims, and thus, may not correspond to clinical diagnoses, introducing potential misclassification. Occasionally, diagnosis codes persist in the records long after the clinical condition has been resolved or improved. Additionally, the data were not originally collected for research purposes; therefore, some values were missing. Finally, since the database was anonymized for medical institutions, no follow-up data were available after the individuals were transferred to other healthcare facilities, which may have caused an underestimation of the outcomes considered in this study. Although we assumed unique inclusion of individuals, re-registration at other centers after transfer may have introduced duplicate records.

## Supplementary Information

Below is the link to the electronic supplementary material.

Supplementary file1 (DOCX 38 kb)

## Data Availability

The datasets used in this study are not publicly available because the data were obtained from JMDC Inc. The data will be available on reasonable request to the corresponding author with the permission of JMDC Inc.

## Abbreviations

ATC: Anatomical therapeutic classification (WHO)
CSP: Cyclosporin
cGVHD: Chronic graft-versus-host disease
GVHD: Graft-versus-host disease
HCT: Hematopoietic cell transplantation
ICD-10: International classification of diseases 10th revision
IQR: Interquartile range
MMF: Mycophenolate mofetil
RC: Receipt code
SD: Standard deviation
TAC: Tacrolimus

## References

[1] Baumrin E, Loren AW, Falk SJ, Mays JW, Cowen EW. Chronic graft-versus-host disease. Part I- epidemiology, pathogenesis, and clinical manifestations. J Am Acad Dermatol. 2024;90:1–16. 10.1016/j.jaad.2022.12.024 PMC10287844 36572065

[2] Martin PJ, Carpenter PA, Sanders JE, Flowers ME. Diagnosis and clinical management of chronic graft-versus-host disease. Int J Hematol. 2004;79:221–8. 10.1532/ijh97.03176. 15168588

[3] Wolff D, Lawitschka A. Chronic graft-versus-host disease. In: The EBMT handbook. Cham: Springer International Publishing; 2019. p. 331–45. 10.1007/978-3-030-02278-5_44.

[4] Martelin E, Volin L, Itälä-Remes M, Niittyvuopio R, Lindström V, Heiskanen J, et al. Incidence and risk factors of secondary cancers after allogeneic stem cell transplantation: analysis of a single centre cohort with a long follow-up. Bone Marrow Transplant. 2019;54:334–7. 10.1038/s41409-018-0290-6. 30104720

[5] Yokota A, Ozawa S, Masanori T, Akiyama H, Ohshima K, Kanda Y, et al. Secondary solid tumors after allogeneic hematopoietic SCT in Japan. Bone Marrow Transplant. 2012;47:95–100. 10.1038/bmt.2011.23. 21358690

[6] Nagai K, Tanaka T, Kodaira N, Kimura S, Takahashi Y, Nakayama T. Data resource profile: JMDC claims database sourced from health insurance societies. J Gen Fam Med. 2021;22:118–27. 10.1002/jgf2.422. PMC8090843 33977008

[7] Hupfeld CJ, Iñiguez-Lluhi JA. Chapter 50 Adrenocorticotropic hormone, adrenal steroids, and the adrenal cortex. In: Brunton LL, Knollmann BC, editors. Goodman and Gilman’s the pharmacological basis of therapeutics. New York: McGraw-Hill; 2022.

[8] Bachier CR, Aggarwal SK, Hennegan K, Milgroom A, Francis K, Dehipawala S, et al. Epidemiology and treatment of chronic graft-versus-host disease post-allogeneic hematopoietic cell transplantation: a US claims analysis. Transplant Cell Ther. 2021;27:504.e1-504.e6. 10.1016/j.jtct.2020.12.027. 34158154

[9] Kanda J, Mitsuyoshi T, Sakurai M, Nishimori H, Murata M, Uchida N, et al. Real-world outcomes of graft-versus-host disease after allogeneic hematopoietic stem cell transplantation in Japan: retrospective analysis of the Transplant Registry Unified Management Program registry. Transplant Cell Ther. 2024;30:907.e1-907.e16. 10.1016/j.jtct.2024.06.023. 38960321

[10] Kanda J, Nakasone H, Atsuta Y, Toubai T, Yokoyama H, Fukuda T, et al. Risk factors and organ involvement of chronic GVHD in Japan. Bone Marrow Transplant. 2014;49:228–35. 10.1038/bmt.2013.151. 24076549

[11] Ponce DM, Politikos I, Alousi A, Carpenter PA, Milano F, MacMillan ML, et al. Guidelines for the prevention and management of graft-versus-host disease after cord blood transplantation. Transplant Cell Ther. 2021;27:540–4. 10.1016/j.jtct.2021.03.012. 34210500

[12] Zhu X, Tang B, Sun Z. Umbilical cord blood transplantation: still growing and improving. Stem Cells Transl Med. 2021;10(Suppl 2):S62–74. 10.1002/sctm.20-0495. PMC8560197 34724722

[13] Sanda K, Fuji S, Tada Y, Shingai Y, Kasahara H, Yuda S, et al. Impact of stem cell source on secondary steroid for chronic GVHD after allogeneic hematopoietic cell transplantation. Int J Hematol. 2024. 10.1007/s12185-024-03866-1. 39585531

[14] Chakkera HA, Mandarino LJ. Calcineurin inhibition and new-onset diabetes mellitus after transplantation. Transplantation. 2013;95:647–52. 10.1097/TP.0b013e31826e592e. PMC3884894 23076551

[15] M.H. Jagasia, H.T. Greinix, M. Arora National Institutes of Health Consensus Development Project on Criteria for Clinical Trials in Chronic Graft-versus-Host Disease- I. The 2014 Diagnosis and Staging Working Group report. Biol Blood Marrow Transplant. 2015;21 10.1016/j.bbmt.2014.12.001 PMC4329079 25529383

