# Supplementary material for: Real-world treatments and outcomes of chronic graft-versus-host disease after allogeneic hematopoietic stem cell transplantation: a Japanese claims analysis
Source: Int J Hematol. 2026 May 27;124(4):580–9. doi: 10.1007/s12185-026-04228-9 (PMC13638707; doi:10.1007/s12185-026-04228-9)
Supplement: Supplementary file 1 [file 12185_2026_4228_MOESM1_ESM.docx]

**SUPPLEMENTARY TABLES 1–7**

Supplementary Table S1. Definition of inclusion and exclusion criteria

| Item | | Source | Code (in regular expression) |
| --- | --- | --- | --- |
| Inclusion criteria | |  |  |
|  | GVHD | ICD-10 | T860 (Complications of bone marrow transplant) |
|  |  | RC^*^ | 884363[78] |
|  | Chronic GVHD | RC | 8846345 |
|  | Hematopoietic stem cell transplantation | RC | 1502(25910\|97810)\|150349810 |
| Exclusion criteria | |  |  |
|  | Autotransplantation |  | 150266[34]10 |
|  | post-transfusion GVHD | RC | 8841395 |

^*^Receipt code

Supplementary Table S2. Definition of covariates

| Item | | Source | Code (in regular expression) |
| --- | --- | --- | --- |
| Primary disease | | | |
|  | Hodgkin lymphoma | ICD-10 | C81[179] |
|  | non-Hodgkin lymphoma | ICD-10 | C8(2[39]\|3[0357]\|4[4568]\|\|5[19]\|6[0125] |
|  | Myeloma and primary macroglobulinemia | ICD-10 | C880\|C90[01] |
|  | Lymphocytic leukemia | ICD-10 | C91[013679] |
|  | Adult T-cell leukemia | ICD-10 | C915 |
|  | Myelogenous leukemia | ICD-10 | C92[013-79]\|C93[013] |
|  | Myelodysplastic syndrome | ICD-10 | D46[245679] |
|  | Myeloproliferative disorder | ICD-10 | D47[14] |
|  | Aplastic anemia | ICD-10 | D61[0239] |
|  | Others | ICD-10 | C795\|C94[67]\|C95[019]\|D595\|D71\|D761\|D823 |
| Source of hematopoietic stem cells | | | |
|  | Bone marrow | RC | 150225910 |
|  | Peripheral blood | RC | 150297810 |
|  | Umbilical cord blood | RC | 150349810 |
| Unrelated donor | | RC | 150405070 |
| Drug | | | |
|  | Cyclosporin | ATC | L04AD01 |
|  | Tacrolimus | ATC | L04AD02 |
|  | Steroids | ATC | H02A(A02\|B(0[124689]\|10)) |
|  | Mycophenolate mofetil | ATC | L04AA06 |
|  | Others | ATC | L04AA04\|L01EL01\|D11AH09 |
|  |  | RC | 629700101 |

Supplementary Table S3. Definition of prednisolone-equivalent dose

| Glucocorticoid | ATC code | Equivalent dose (mg) |
| --- | --- | --- |
| Cortisone | H02AB10 | 25 |
| Hydrocortisone^*^ | H02AB09 | 20 |
| Prednisolone | H02AB06 | 5 |
| Methylprednisolone | H02AB04 | 4 |
| Triamcinolone | H02AB08 | 4 |
| Betamethasone | H02AB01 | 0.75 |
| Dexamethasone^*^ | H02AB02 | 0.75 |
| Fludrocortisone | H02AA02 | 2 |
| ^*^Multiplied by 1.3 in case of oral form. | | |

Supplementary Table S4. Definition of complications and anti-infective agents

| Item | | Source | Code (in regular expression) |
| --- | --- | --- | --- |
| Complication | | | |
|  | Cytomegalovirus infection | ICD-10 | B25[0-9] |
|  | Erythropenia (anemia) | ICD-10 | D59[1-9]\|D6[0-4] |
|  | Pancytopenia | ICD-10 | D61[0-9] |
|  | Thrombopenia | ICD-10 | D69[3-6] |
|  | Neutropenia | ICD-10 | D70\|D728 |
|  | Hypogammaglobulinemia | ICD-10 | D80[0-7] |
|  | Hypothyroidism | ICD-10 | E03[0-9] |
|  | Diabetes mellitus | ICD-10 | E1[0-4] |
|  | Growth hormone deficiency | ICD-10 | E230 |
|  | Dry eye | ICD-10 | H041 |
|  | chronic obstructive pulmonary disease | ICD-10 | J4[2-4] |
|  | Bronchial asthma | ICD-10 | J4[5-6] |
|  | Osteoporosis | ICD-10 | M8[0-1] |
| Anti-infective agent | | | |
|  | Intravenous broad-spectrum penicillins | ATC | J01C2 |
|  | Oral cephalosporins | ATC | J01D1 |
|  | Intravenous cephalosporins | ATC | J01D2 |
|  | Trimethoprim preparations | ATC | J01E |
|  | Macrolide antibiotics | ATC | J01F |
|  | Oral fluoroquinolones | ATC | J01G1 |
|  | Penem antibiotics | ATC | J01P2 |
|  | Glycopeptide antibiotics | ATC | J01X1 |
|  | Systemic antifungal agents | ATC | J02A |
|  | Anti-herpesvirus agents | ATC | J05B3 |
|  | Intravenous immunoglobulins | ATC | J06C |

Supplementary Table S5. Featured patient characteristics stratified by age

Age <15	Age 15–54	Age ≥55

*N* = 138	*N* = 665	*N* = 287

Characteristic (selected)	*n*	%	*n*	%	*n*	%

Primary disease	lymphocytic leukemia	34	24.6	163	24.5	48	16.7

myeloid leukemia	29	21.0	274	41.2	138	48.1

myelodysplastic syndrome	19	13.8	94	14.1	99	34.5

HCT^*^ source	bone marrow	54	39.1	187	28.1	81	28.2

peripheral blood	15	10.9	162	24.4	78	27.2

umbilical cord blood	58	42.0	210	31.6	87	30.3

Observation period	median (months)	34.5	17	10

60 months or more	39	28.3	107	16.1	13	4.5

^*^Hematopoietic cell transplant

Supplementary Table S6. The first-line immunosuppressants selected in each age group

Age <15	Age 15–54	Age ≥55

*N* = 107 (78%)^*^	*N* = 547 (82%)^*^	*N* = 231 (81%)^*^

*n*	%	Days^**^	*n*	%	Days^**^	*n*	%	Days^**^

TAC + Steroid	45	42.1	111	149	27.2	81	79	34.2	109

TAC	34	31.8	42	144	26.3	46	66	28.6	65.5

Steroid	13	12.1	13	110	20.1	16.5	56	24.2	14

CSP	3	2.8	126	65	11.9	50	6	2.6	111

CSP + Steroid	2	1.9	56	41	7.5	140	15	6.5	63

TAC + Steroid + MMF	6	5.6	450	17	3.1	224	4	1.7	116

^*^The number of patients (and the percentage) who received the line of treatment at the stage.

^**^Median days of continuous dosing are shown.

Abbreviations: TAC, tacrolimus; CSP, cyclosporin; MMF, mycophenolate mofetil.

Supplementary Table S7. Use of immunosuppressants and the death of cGVHD patients

All patients	Any IMS^*^ prescribed

*N* = 1,090	*N* = 885

*n*	%	*n*	%

Deceased during the observation period	334	30.6	(100)	281	31.8	(100)

On the day 1–89	127	11.7	(38.0)	165	18.6	(58.7)

On the day 90–179	71	6.5	(21.3)	48	5.4	(17.1)

On the day 180–269	32	2.9	(9.6)	22	2.5	(7.8)

On the day 270–359	23	2.1	(6.9)	11	1.2	(3.9)

On the day ≥360	81	7.4	(24.2)	35	4.0	(12.5)

^*^Immunosuppressant
